# University Day

**Published:** 1911-03

**Authors:** 


### University Day

THE university day exercises of the University of Buffalo were held on Tuesday, February 22, 1911, at the Teck Theater. The dominant note of this year's celebration was a greater university.

Reverend Frank S. Fitch opened the exercises with prayer and after the singing of the Alma Mater, Chancellor Charles P. Norton introduced Professor Frank S. Fosdick as the orator of the day. Mr. Fosdick's address was on Civic Upbuilding.

The influences which make and unmake nations, he said, have the same potency in cities. After specifying several civic problems confronting the city of Buffalo, Mr. Fosdick said:

Now if I were to hazard an opinion as to the reason for delay in all these important matters, I should say emphatically that what Buffalo needed more than anything else was a vigorous, intelligent moral public spirit that would so pervade the body politic as to force by its very momentum the speedy settlement of all things vital to the municipality and would be sure that they are settled right. The greatest agency in accomplishing this end is present educational system by a free university, the greater University of Buffalo, and the city's greatest problem is how to accomplish this quickly and effectively. Nearly 4,000 persons have been graduated from the present departments. All honor to them. But it seems that a fitting time has come to broaden the scope of its work and to organize a purely collegiate department where our boys and our girls can freely obtain what they desire so much. A site has been purchased by the university, and an enabling act was passed last year by the legislature authorising the city of Buffalo to enter into contract with the University of Buffalo for the purpose of providing for the free higher education of the inhabitants of the city and making it permissible for Buffalo to appropriate annually a sum not exceeding $75,000 for the establishment and maintenance of a college in the university.

Here, then, is the situation: We have a site, we have an enabling act of the legislature, but what about suitable buildings and other appurtenances? Where are they to be obtained? I wish today, more than ever before, that riches were mine that I might have the great privilege of giving freely toward this movement that should appeal to the civic patriotism of all of us. In behalf of the many boys and girls who are longing for college advantages and whose future usefulness hangs in the balance; in behalf of so many who have become stunted and dwarfed from arrested development for which they are blameless; in behalf of the fathers and mothers who would gladly make daily and hourly sacrifices if only their sons and daughters might have the benefits of higher education, and lastly in behalf of our loved city itself which needs the infusion of well-equipped, well-balanced minds into its activities and the civic upbuilding that can come only from upright and virile citizenship I plead for a free university that shall be a fitting crown to Buffalo's greatness."

